# Pediatric tumefactive multiple sclerosis case (with baló-like lesions), diagnostic and treatment challenges

**DOI:** 10.1007/s10072-022-06396-y

**Published:** 2022-09-29

**Authors:** Christina Kamari, Emmanouil Galanakis, Maria Raissaki, George Briassoulis, Georgia Vlachaki, Pelagia Vorgia

**Affiliations:** 1 Pediatric Department, General Hospital of Kastoria, Kastoria, Greece; 2 Department of Paediatrics, University of Crete, Heraklion, Greece; 3 Department of Radiology, Medical School, University Hospital of Heraklion, University of Crete, Heraklion, Greece; 4 Pediatric Intensive Care Unit, School of Medicine, University of Crete, Heraklion, Greece; 5 Pediatric Department, Venizelion General Hospital Heraklion, Heraklion, Crete, Greece; 6 Agri-Food and Life Sciences Institute, University Research Center, Hellenic Mediterranean University, 105 Isavron street, 71303 Heraklion, Crete, Greece

**Keywords:** Tumefactive Multiple sclerosis, Baló concentric sclerosis, Pediatric multiple sclerosis, Fingolimod

## Abstract

**Background:**

Multiple sclerosis (MS) is a demyelinating disease of the central nervous system, rare during childhood. MS variations, like tumefactive MS and Balo concentric sclerosis, constitute puzzling to treat diagnostic dilemmas for pediatric patients. Differential diagnosis, mainly from brain tumors, is an absolute necessity. In addition, apart from treating acute attacks, immunomodulatory alternatives are limited.

**Case:**

We present a 12.5-year-old boy diagnosed, 5 years ago, with tumefactive relapsing–remitting MS, with severe recurrent clinical attacks. Definite diagnosis of demyelination was achieved via combined brain imaging including magnetic resonance (MR) imaging, MR spectroscopy and computed tomography, avoiding brain biopsy. Acute attacks showed satisfactory response to aggressive treatment choices, like plasmapheresis and cyclophosphamide, but age-appropriate immunomodulating treatment was available, only 2 years later. Finally, after a last radiological relapse, when he was 10 years old, fingolimod was initiated. He has been clinically and radiologically stable since, presenting an excellent treatment tolerance.

Multiple sclerosis (MS) is a demyelinating disease of the central nervous system with an incidence of 0.2–2.9 per 100,000 children worldwide, of which 0.3% are younger than 10 years at diagnosis^1^. Tumefactive MS (TMS) and Baló’s concentric sclerosis are rarely referred in the literature of MS variants. They are challenging to diagnose and treat situations that should usually be differentiated from brain tumors, especially in children^2,3^.

We present a 7.5-year-old boy with TMS and a Baló-like lesion who initially presented with subacute right hemiparesis, ipsilateral facial palsy, and an otherwise unremarkable personal and family history. Brain magnetic resonance imaging (MRI) revealed three tumefactive (over 2 cm) periventricular lesions. One tumefactive lesion exhibited a concentric ring appearance, an open ring diffusion restriction pattern, and thin ring enhancement, consistent with a Baló-like lesion (Fig. 1A, 1B). Spinal MRI was normal. Laboratory testing excluded autoimmune diseases and infection (Table 1). The absence of cortical involvement, mass effect, and seizures in combination with the aforementioned lesions at presentation favored the diagnosis of clinically isolated syndrome^4^. The patient was treated with methylprednisolone pulses, intravenous immunoglobulins, and finally showed optimal response to plasmapheresis.

**Fig. 1:**
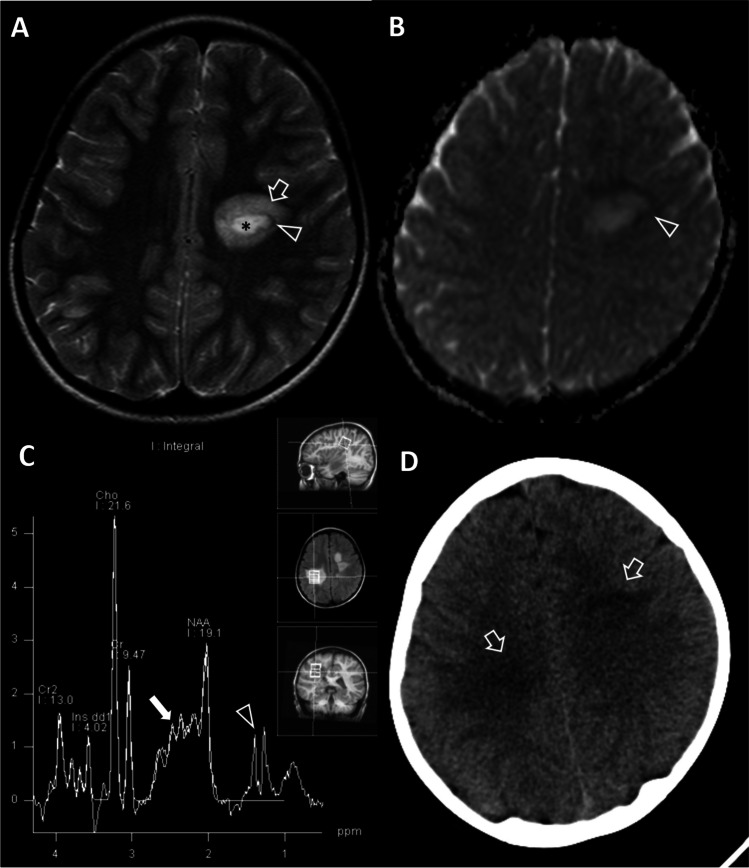
Brain imaging. **A** Axial T2-weighted image at diagnosis, showing a left-sided tumefactive hyperintense lesion (*) surrounded by an internal hypointense (arrowhead) and external hyperintense ring (open arrow). This concentric ring appearance is attributed to areas of demyelination alternating with relatively preserved myelin (Baló-like tumefactive lesion). **B** Diffusion (B 800) image shows an open ring of restricted diffusion (arrowhead). **C** Proton MR spectroscopy with voxel localization over a new tumefactive right-sided lesion. There is an elevation of the β,γ-Glx peaks at 2.1–2.6 ppm region (arrows) and presence of lactate doublet (arrowhead) that suggest an inflammatory demyelinating process. Α decrease of N-acetylaspartate (NAA), increased choline (Cho) are non-discriminating findings. **D** CT shows hypoattenuation at the areas of known white matter lesions (arrows)

**Table 1:** Laboratory findings at diagnosis and five months later with appearance of new tumefactive lesions. *CSF*, cerebrospinal fluid; *ACE*, angiotensin converting enzyme; *Anti-NMO*, antibodies against astrocytes, anti-aquaporin-4; *Anti-MOG*, antibodies against Myelin oligodendrocyte glycoprotein

Laboratory findings at diagnosis		Laboratory findings with new tumefactive lesions appearance
Basic hematologic and biochemical testing, vitamin B12, folic acid, 25(OH) vitamin D3	Normal 25(OH) Vitamin D3: low (supplementation)	Normal
Infectious disease screening*	Negative	Negative
Lumbar puncture	5 white blood cells, 0 red blood cells, glucose 64 mg/dl, Glu CSF/serum ratio 0.64, CSF protein 39.5 mg/dl, CSF culture sterile, CSF PCR HSV1&2 (-) CSF cytology negative	2 white blood cells, 0 red blood cells, glucose 64 mg/dl, Glu CSF/serum ratio 0.8, **CSF protein 47.5 mg/dl** CSF culture sterile CSF cytology negative
IgG index/oligoclonal bands	− 3.19/absence	** + 3.36**/**mirror pattern**
Immunological screening**/ACE	Negative/normal	Negative/normal
Anti-NMO, anti-MOG	Negative at serum and CSF	Negative at serum and CSF

Infectious disease screening*: infection biomarkers (C-reactive protein, ferritin, erythrocyte sedimentation rate), blood culture, serology screening for HIV, HAV, HBV, HCV, EBV, CMV, ADV, measles, mumps, rubella, influenza, parainfluenza, HSV, Coxsackie, Bartonella, mycoplasma, tuberculin skin test. Immunological Screening**: antiphospholipid antibodies, rheumatoid factor, antinuclear antibodies, anti-double-stranded DNA antibodies, extractable nuclear antigen (ENA) panel, anti-neutrophil cytoplasmic antibodies

One month later, the patient developed left hemiparesis which gradually evolved into motor disability, while new lesions at the right periventricular white matter (Fig. 1C) and at additional sites were consistent with MS, according to space and time international criteria^4^. Patient responded considerably to a five, every other day, cyclophosphamide infusion scheme of 800 mg/m^2^ body surface area. Despite clinical improvement with mild residual neurological deficit, in view of new lesion development and inconclusive laboratory investigations (Table 1), a brain tumor should have actively been excluded. Magnetic resonance spectroscopy (MRS) revealed elevated β,γ-Glx peaks and a lipid-lactate doublet, which favored the diagnosis of demyelination. Brain biopsy was avoided due to invasiveness and possibility of misleading results^5^. Instead, computed tomography (CT) revealed hypoattenuation at the areas of abnormality (Fig. 1D), excluding cerebral lymphoma and supporting the diagnosis of TMS^6^. New MRI lesions prompted treatment with methylprednisolone pulses which resulted in clinical stability.

At that time, considering the absence of evidence-based guidelines for TMS immunomodulating treatment, appropriate for our patients’ age, a “wait and see” close follow-up approach was chosen^1,2,4^. Treatment-free he remained clinically (Extended Disability Status Scale: 1) and radiologically stable for almost 2 years when a large MRI non-enhancing lesion appeared. Meeting age criteria, treatment with fingolimod was immediately initiated, as it was finally approved as an oral therapy for highly active relapsing–remitting forms of MS (RRMS) for children over 10 years (EMA/685570/2018, EMEA/H/C/002202). Patient exhibited excellent tolerance; he is clinically and radiologically stable, 2 years later.

Differential diagnosis from brain tumors is challenging. Brain biopsy is useful^6^ but requires a high level of expertise; otherwise, results may be misleading. Multimodality imaging with MRI, MRS, and CT, along with clinical findings, has a diagnostic accuracy of up to 97%^5^. The absence of cortical involvement, seizures, cerebral mass effect and the relapsing clinical course responding to treatment make the diagnosis of demyelination practically non-questionable.

Regarding treatment, these typical RRMS patients respond to aggressive therapeutic options, like plasmapheresis and cyclophosphamide, as occurred in our patient, and finally require disease-modifying therapy. A 2-year treatment with fingolimod proved an effective and safe treatment choice for this child.

Conclusively, the diagnosis of MS variants should be facilitated by relevant accessible diagnostic tools. Brain biopsy remains an option, although invasive. Immunomodulating therapy, like fingolimod, appears as a safe, effective option for children with newly diagnosed tumefactive RRMS. To our knowledge, this is the first child diagnosed 5 years ago, finally treated with fingolimod, and followed under this treatment up for 2 years, described in the literature.
