# Correction to: Pediatric tumefactive multiple sclerosis case (with baló‑like lesions), diagnostic and treatment challenges

**DOI:** 10.1007/s10072-022-06502-0

**Published:** 2022-11-17

**Authors:** Christina Kamari, Emmanouil Galanakis, Maria Raissaki, George Briassoulis, Georgia Vlachaki, Pelagia Vorgia

**Affiliations:** 1 Pediatric Department, General Hospital of Kastoria, Kastoria, Greece; 2 Department of Paediatrics, University of Crete, Heraklion, Greece; 3 Department of Radiology, Medical School, University Hospital of Heraklion, University of Crete, Heraklion, Greece; 4 Pediatric Intensive Care Unit, School of Medicine, University of Crete, Heraklion, Greece; 5 Pediatric Department, Venizelion General Hospital Heraklion, Heraklion, Crete Greece; 6 Agri‑Food and Life Sciences Institute, University Research Center, Hellenic Mediterranean University, 105 Isavron street, 71303 Heraklion, Crete Greece

**Correction to: Neurological Sciences (2022)**

**https://doi.org/10.1007/s10072-022-06396-y**

Originally, the article was published with inverted author names. The author names are now correctly cited.

The original article has been corrected.

